# Lung Recruitment Strategies During High Frequency Oscillatory Ventilation in Preterm Lambs

**DOI:** 10.3389/fped.2018.00436

**Published:** 2019-01-22

**Authors:** Martijn Miedema, Karen E. McCall, Elizabeth J. Perkins, Regina B. Oakley, Prue M. Pereira-Fantini, Anushi E. Rajapaksa, Andreas D. Waldmann, David G. Tingay, Anton H. van Kaam

**Affiliations:** ^1^Neonatal Research, Murdoch Children's Research Institute, Melbourne, VIC, Australia; ^2^Department of Neonatology, Emma Children's Hospital, Amsterdam UMC, University of Amsterdam, Amsterdam, Netherlands; ^3^School of Medicine and Medical Science, University College Dublin, Dublin, Ireland; ^4^Department of Neonatology, Royal Children's Hospital, Melbourne, VIC, Australia; ^5^Department of Paediatrics, University of Melbourne, Melbourne, VIC, Australia; ^6^Swisstom AG, Landquart, Switzerland

**Keywords:** preterm infant, mechanical ventilation, high frequency ventilation, neonate, respiratory distress syndrome, ventilator induced lung injury

## Abstract

**Background:** High frequency oscillatory ventilation (HFOV) is considered a lung protective ventilation mode in preterm infants only if lung volume is optimized. However, whilst a “high lung volume strategy” is advocated for HFOV in preterm infants this strategy is not precisely defined. It is not known to what extent lung recruitment should be pursued to provide lung protection. In this study we aimed to determine the relationship between the magnitude of lung volume optimization and its effect on gas exchange and lung injury in preterm lambs.

**Methods:** 36 surfactant-deficient 124–127 d lambs commenced HFOV immediately following a sustained inflation at birth and were allocated to either (1) no recruitment (low lung volume; LLV), (2) medium- (MLV), or (3) high lung volume (HLV) recruitment strategy. Gas exchange and lung volume changes over time were measured. Lung injury was analyzed by post mortem pressure-volume curves, alveolar protein leakage, gene expression, and histological injury score.

**Results:** More animals in the LLV developed a pneumothorax compared to both recruitment groups. Gas exchange was superior in both recruitment groups compared to LLV. Total lung capacity tended to be lower in the LLV group. Other parameters of lung injury were not different.

**Conclusions:** Lung recruitment during HFOV optimizes gas exchange but has only modest effects on lung injury in a preterm animal model. In the HLV group aiming at a more extensive lung recruitment gas exchange was better without affecting lung injury.

## Introduction

Despite the increasing use of non-invasive respiratory support ~45% of preterm infants < 28 weeks of gestation still require invasive mechanical ventilation (1). Although mechanical ventilation improves respiratory function it often results in secondary ventilator induced lung injury (VILI). VILI is considered one of the major risk factors in the development of bronchopulmonary dysplasia (BPD) (2, 3).

Alveolar overdistension resulting from large tidal volumes (volutrauma) during mechanical ventilation has been identified as a risk factor for VILI. For this reason, high-frequency oscillatory ventilation (HFOV), which by design delivers tidal volumes smaller than dead space, is considered a lung protective ventilation mode (4, 5). HFOV is now most often used as a rescue therapy in preterm infants when other modalities have failed or create unacceptable injury potential (6). However, lung protection during HFOV can only be obtained if existing atelectasis is reversed. This has been shown in the meta-analysis of large trials of preterm infants receiving first-intention HFOV, which conclusively show that a high-lung volume strategy is needed for HFOV to be safe and efficacious (7, 8). A limitation of these meta-analyses has been providing clinicians with a clear definition of a high-lung volume strategy. This has led to considerable variation in clinical practice, which may explain the discrepancy in lung protective benefit between animal studies and randomized trials (4, 7–10).

The original evidence demonstrating the need for a high-lung volume strategy during HFOV arose from a single animal study using an adult rabbit saline lung lavage model of surfactant deficiency (4). In this pivotal study, McCulloch et al. showed that applying HFOV with a higher end expiratory lung volume (EELV) resulted in less lung injury than HFOV with lower EELV (5). Notwithstanding the impact this study has had on HFOV clinical evolution, lung injury initiation, and consequences are different in the developmentally immature preterm lung. Furthermore, lung volume was not measured in this study, with volume defined by oxygen response, a common clinical practice now. As such it is unknown to what volume extent a high-lung volume strategy, and by extension clinical strategy of lung recruitment, should be pursued: if EELV is too low residual atelectasis will persist, with overdistension occurring if too high.

The aim of this study is to determine the effect of different levels of lung volume stabilization, specifically a high and moderate lung volume, on gas exchange and initiation of VILI when compared to a low volume strategy without any intentional recruitment in high-frequency ventilated preterm lambs. We hypothesize that a higher recruitment level will result in less residual atelectasis, better gas exchange, and less lung injury.

## Materials and Methods

The study was performed at the animal research facility of the Murdoch Children's Research Institute, Melbourne, Australia and approved by the Institution's Animal Ethics Committee in accordance with guidelines of the National Health and Medical Research Council of Australia.

### Animal Preparation

124–127 d preterm lambs (term 142 d and equivalent to preterm infants with a gestational age of 24–27 weeks) were delivered via cesarean section under general anesthesia to date-mated Border-Leicester ewes who received 11.5 mg betamethasone 24 and 48 h prior to delivery (11, 12). Where possible twin pregnancies were used, and lambs were randomly allocated before birth to ensure equal paired-permutations of the three ventilation groups. After delivery of the fetal head and chest, lambs were instrumented as previously described (13, 14). This included catheterizing the carotid artery and external jugular vein, intubation with a 4.0 cuffed endotracheal tube (ETT) and passively draining lung liquid (11, 15). Immediately prior to delivery a custom made 32 electrode electrical impedance tomography (EIT) lamb belt (Swisstom AG, Lanquart, Switzerland) was placed around the chest and a 10 s reference EIT recording of the unaerated lung made (11). Anesthesia and analgesia was initiated with ketamine and midazolam intravenously at levels that also suppressed spontaneous breathing throughout the study period. After delivery, the lamb was weighted and placed in supine position on a neonatal resuscitation table before the ventilation protocol was initiated (11, 14).

### Ventilation Protocol

**Figure 1:**
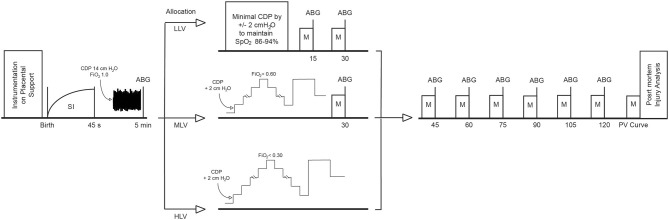
Overview of the experimental protocol. HFOV is initiated directly after the SI. ABG, arterial blood gas; CDP, continuous distending pressure; HLV, high lung volume; LLV, low lung volume; M, measurement of physiological, ventilator, haemodynamic, and EIT parameters, MLV, medium lung volume, SI, sustained inflation.

Figure 1 details the ventilation protocol. At birth all animals received a sustained inflation of 35 cmH_2_O over 45 s with a fraction of inspired oxygen (FiO_2_) of 1.0 using Neopuff™ T-Piece resuscitator (Fisher and Paykel, Health Care, Auckland, New Zealand) to aerate the fluid-filled lung and establish a functional residual capacity. This was repeated at 40 cm H_2_O if an insufficient response, defined as not obtaining a plateau in lung volume during the SI, was seen in EELV measured real time using the Swisstom Pioneer EIT system (11, 14). Then HFOV (Sensormedics 3100B, Carefusion, Yorba Linda, CA) was commenced at a continuous distending pressure (CDP) of 14 cmH_2_O, frequency of 10 Hz, inspiration time of 33%, pressure amplitude of 45 cmH_2_O, and a FiO_2_ 1.0 (16). After 5 min of HFOV at these settings animals commenced the allocated ventilation strategy:

**(1) Low lung volume strategy (LLV):** HFOV at CDP 14 cmH_2_O without any lung recruitment. This represented the minimum CDP found to be tolerated in our previous HFOV study. CDP was only changed (in 2 cm H_2_O in- or decrements) if preductal peripheral oxygen saturation (SpO_2_) was outside the 88–94% target range in FiO_2_ of 1.0.

**(2) Medium lung volume strategy (MLV):** An oxygenation guided lung recruitment strategy aiming to maintain the SpO_2_ between 88 and 94% in FiO_2_ ≤ 0.60. Briefly, the CDP was stepwise increased with 2 cm H_2_O every 2–3 min to recruit atelectatic lung units. If oxygenation improved due to the reduced intrapulmonary shunt, the FiO_2_ was weaned in steps of 0.1 every 2 min. The CDP was increased until the FiO_2_ was ≤ 0.60, termed the openings pressure (CDPo) of the lung. Next, the CDP was reduced by 2 cm H_2_O every 2–3 min to identify the closing pressure (CDPc) defined as CDP in which SpO_2_ fell below 88% at this FiO_2_. The lung was re-opened with the known CDPo for 4–5 min and HFOV continued at the final CDP (CDPf) defined as CDPc +2 cm H_2_O (9, 10, 17).

**(3) High lung volume strategy (HLV):** An oxygenation guided recruitment strategy aiming to maintain 88–94% SpO_2_ target in FiO_2_ <0.30. The method of determining CDPo, CDPc, CDPf was the same as MLV except now a FiO_2_ <0.30 was targeted. This was the FiO_2_ that could be achieved in 98% of preterm infants managed with a similar lung recruitment strategy (17).

In all three groups, CDP increases were limited to 30 cm H_2_O as a previous study from our group confirmed that higher CDPs resulted in significant hemodynamic compromise in this lamb population (16).

Following each strategy, all lambs were oscillated at CDPf for a total of 120 min and the pressure amplitude adjusted to maintain PaCO_2_ within the normal range (35–45 mmHg). The CDP was increased in 2 cmH_2_O pressure steps every 2–5 min if the SpO_2_ fell below 88%, and similarly decreased if SpO_2_ was >94%. Exogenous surfactant was intentionally not administered as prophylactic or early treatment is no longer advocated. Furthermore, several groups have adopted an approach to first recruit the lung and then administer surfactant if indicated (17, 18). After 120 min, the FiO_2_ was increased to 1.0 for 3 min, the lambs killed by a lethal intravenous dose of pentobarbitone and the ETT disconnected to atmosphere. An additional fifteen age-matched fetuses were also euthanized at delivery to act as an unventilated control (UVC) group for lung injury analysis (12).

### Physiological Data Collection and Analysis

Preductal SpO_2_, heart rate, arterial blood pressure, rectal temperature (HP48S, Hewlett Packard, Andover, USA), airway opening pressure and flow (Florian Respiratory Mechanics monitor, Acutronic AG, Hirzel, Switzerland) and cerebral blood flow (Transonic ultrasonic flow probe Transonic System, Inc., Ithaca, USA) were continuously monitored from birth and recorded in 15 min intervals into a custom built LabChart template (AD instrument, Colorado Springs, USA) (19). Arterial blood gasses analyses were performed at 5 min after birth, at every pressure step of the lung recruitment procedure and then 15 min during the 120-min ventilation period.

An *in vivo* post mortem pressure-volume curve was reconstructed immediately using the super syringe technique to maximum pressure of 35 cmH_2_O (termed total lung capacity; TLC). Static maximal lung compliance (*C*_max_) was calculated from the steepest part of the deflation limb of the pressure-volume relationship (20).

### Pressure and Time Related Analysis

The individual and summarized relationship between SpO_2_/FiO_2_ ratio and alveolar-arterial oxygen difference (AaDO_2_) vs. CDP and time were determined. To account for different absolute CDPo values, pressure data was standardized for the MLV and HLV groups by referencing CDPo as 0 cm H_2_O.

EIT data was sampled at 48 Hz and recorded using STEM software (Swisstom, Lanquart, Switzerland) with a lamb-specific finite-element model lung reconstruction algorithm (11, 21). From these the end expiratory lung impedance (EELI) changes were extracted and calibrated (ml/kg) using the post mortem pressure-volume curve to generate EELV data (14). The EIT data were used to reconstruct the individual- and summarized pressure-EELV relationships for the OLV groups at the start of the ventilation period and to plot the summarized time-EELV curves for all groups. Regional data were generated for the ventral half of the cross-section (non-dependent lung area) and dorsal halve (dependent lung area).

### Lung Injury Data Collection and Analysis

#### Total Lung Protein

Bronchoalveolar lavage (BAL) of the left lung was performed and the alveolar protein concentrations determined using the Lowry method (22, 23).

#### RNA Preparation and Quantitative PCR

Standardized lung samples of the lower and upper part of the right lower lobe were collected and snap frozen in liquid nitrogen. RNA was extracted from lung tissue using TRIzol (Invitrogen, Carlsbad, CA, USA) and 0.1 ug RNA was reverse-transcribed into complementary DNA. Following previously described methods using GAPDH as reference gene, different primers were used to calculate gene expression of connective tissue growth factor (CTGF), cysteine-rich 61 (CYR61), and early growth factor 1 (EGR1) and pro-inflammatory cytokines interleukins 1ß, 6, and 8 as markers of VILI (24, 25).

#### Histological Assessment

For histological evaluation of injury the right upper lung lobe was fixed at 20 cmH_2_O in 4% paraformaldehyde. Next, three standardized gravity dependent regions (lower, middle, and upper region) were cut, sliced, and stained by hematoxylin and eosin (H&E) for microscopic VILI scoring. Five fields of view (FOV)/region were photographed (Leica microsystems, Germany) and injury measurements performed using ImageJ software on the following criteria: (1) number of detached epithelial cells (2) percentage of space occupied by airway, (3) percentage lung tissue, (4) alveolar septa thickness, (5) alveolar saccular area, (6) coefficient of variance of alveolar saccular area (14, 15, 26). Criteria 1 is thought to be a marker of exposure to increased stretch resulting in damaged cells, while criteria 2, 5, and 6 can be seen as changes in airway characteristics. Criteria 3 and 4 reflect damage to the lung morphology (27). All lung injury markers were compared between each intervention and to the UVC group.

### Statistical Analysis

GraphPad PRISM 7 (GraphPad Software, San Diego, USA) was used for statistical analysis. Data were expressed as mean and standard deviation or median with interquartile ranges depending on their distribution. Comparative analysis between two groups was done using a *t*-test or Mann-Whitney test, depending on data distribution. For multiple group comparison we used a one-way or two-way ANOVA for repeated measurements with Tukey *post-hoc* test or Kruskal-Wallis test with Dunn's *post-hoc* test as appropriate. A *p* < 0.05 was considered to be significant. Based on our previous studies, a sample size of 8 lambs per group would allow detection of a difference in AaDO_2_ of 100 mmHg assuming standard deviation of 100 mmHg, 80% type 1 error and p 0.05 (11, 12, 14, 15). Our previous experience with histological assessment of injury and total lung protein levels have suggested that samples sizes of 10–12/group and 7–8/group, respectively, are adequate to determine clinically meaningful differences. A final sample of 12–14 lambs/group was studied to ensure at least 10/group completed the entire protocol and could be included for injury analysis.

## Results

### Animal Characteristics

Thirty-six lambs were studied from which six animals developed a clinical significant pneumothorax (4 in LLV, 1 MLV, and 1 HLV groups) and died (pentobarbitone injection) prior to finishing the entire ventilation protocol. These animals were not analyzed for the outcome measures described, as they did not finish the 120 min of ventilation. As a result, thirty lambs (10 in LLV, 10 in MLV, and 10 in HLV) were available for analysis of all pre-specified outcome parameters. There were no differences between the groups' clinical characteristics, although there was a trend to a higher AaDO_2_ at 5 min after birth in the HLV group (Table 1). Cardiovascular data showed no difference in heart rate, mean arterial blood pressure or cerebral blood flow between the three groups (data not shown).

**Table 1:** Study group characteristics.

**Animal characteristics**	**Ventilation strategy**			
	**UVC (*n* = 15)**	**LLV (*n* = 10)**	**MLV (*n* = 10)**	**HLV (*n* = 10)**
Gestational age (days)	126.8 (0.68)	125.7 (1.1)	125.6 (1.4)	125.0 (0.8)
Arterial cord ph	7.30 (0.06)	7.36 (0.04)	7.36 (0.07)	7.39 (0.04)
Arterial cord PaO_2_ (mmHg)	20.2 (2.3)	25.7 (3.5)	23.1 (6.1)	24.3 (1.8)
Drained fetal lung fluid (ml/kg)	–	14.6 (6.8)	19.3 (10.3)	16.3 (6.0)
First born (%)	0 (0)	5 (50)	6 (60)	6 (60)
Birth weight (grams)	3511 (354)	3375 (544)	3222 (551)	3367 (723)
Female (%)	5 (50)	4 (40)	4 (40)	6 (60)
Sustained inflation (n analyzed)	–	(10)	(9)	(9)
Maximum pressure (cmH_2_O)	–	36.0 (2.1)	36.8 (2.4)	37.5 (2.6)
Inflation volume (ml/kg)	–	24.1 (9.2)	23.1 (10.6)	19.2 (7.6)
AaDO_2_	–	581 (71)	579 (83)	609 (13)
CDPo (cmH_2_O)	–	–	23 [18–26]	29 [26–30]^‡^
FiO_2_	–	–	0.55 [0.55–0.57]	0.30 [0.27–0.34]^†^
CDP final (cmH_2_O)	–	18 [11.5–20]	18 [15–20]	22 [20–24]^**^^,^ ^‡^
FiO_2_	–	1.0 [1.0–1.0]	0.55 [0.55–0.57]^*^	0.30 [0.27–0.34]^**^,^†^

*CDPo, openings pressure; FiO_2_o, fractional inspired oxygen at openings pressure; HLV, high lung volume; LLV, low lung volume; MLV, medium lung volume; UVC, unventilated controls. Data are presented as mean with standard deviation or as median with interquartile ranges. Significant difference is tested by one-way ANOVA with Tukey comparison if normal distributed, otherwise by Kruskal-Wallis with Dunn's multi comparison test*.

* *p < 0.05 vs. LLV*.

** *p < 0.01 vs. LLV*.

† *p < 0.05 vs. MLV*.

‡ *p < 0.01 in contrast to MLV*.

### Lung Recruitment Maneuvers

**Figure 2:**
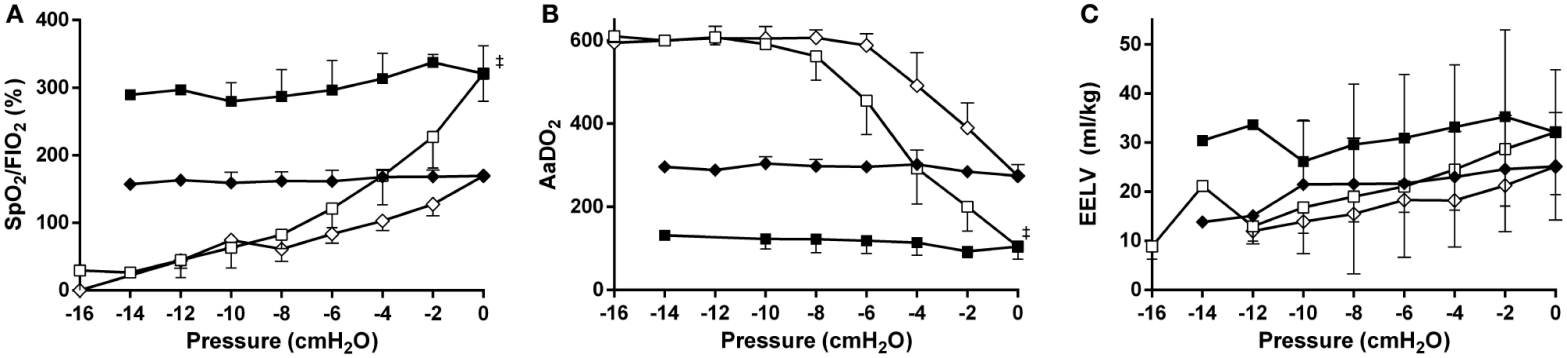
Relationships between mean airway pressure and SpO_2_/FiO_2_ **(A)**, AaDO_2_ **(B)**, and EIT based end-expiratory lung volume (EELV) **(C)** for the medium- (*diamonds*) and the high lung volume recruitment (*squares*) strategies (10 preterm lambs per group). The pressure is related to the openings pressure (Pressure = 0). Recruitment and decruitment phase are indicated in *open* and *closed* symbols, respectively. Data are presented as mean and SD.^‡^*p* < 0.01 in contrast to MLV.

In all MLV and in eight (80%) HLV lambs the targeted reduction in FiO_2_ was obtained, and then maintained to CDPf (Table 1). To do so required a higher CDPo and CDPf in the HLV group. The CDPf was similar in the LLV and MLV groups. At CDPf the FiO_2_ was significantly lower in both recruitment groups compared to LLV group. Both the oxygenation and EELV curves showed hysteresis during the recruitment procedure in the MLV and HLV groups and this was more pronounced in the HLV lambs (Figure 2). As expected, the HLV strategy resulted in a better SpO_2_/FiO_2_ ratio and AaDO_2_ at CDPo and then for all CDP decreases until CDPc (deflation limb) (Figures 2A,B) as compared to the MLV strategy, due to a higher EELV (Figure 2C); mean (SD) 30.9 ± 12.9 ml/kg (HLV) vs. 23.5 ± 9.1 ml/kg (MLV) at CDPf. Individual oxygenation and EELV curves are shown in Supplementary Materials 1, 2.

### Post-lung Recruitment Physiological Data

**Figure 3:**
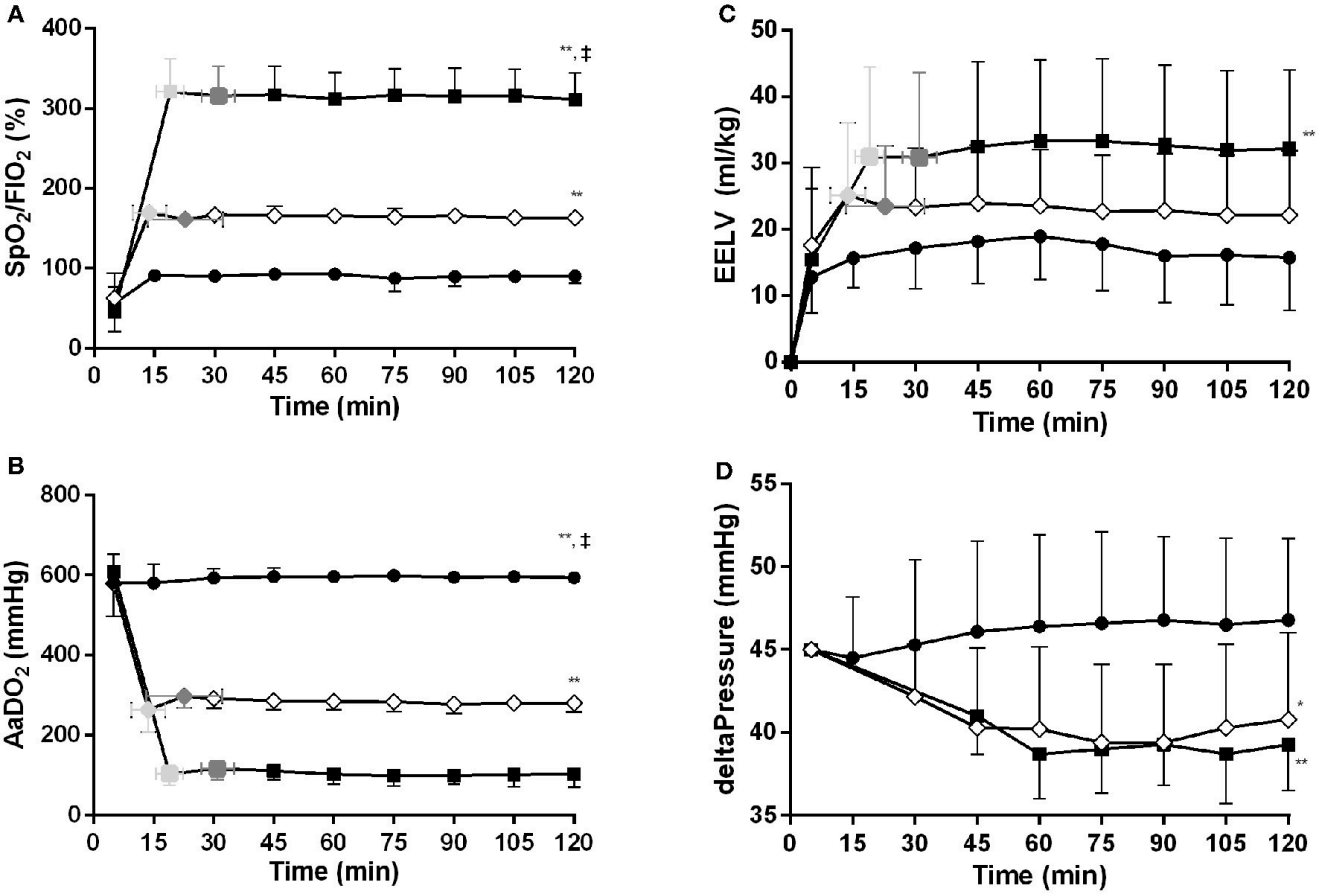
Time vs. SpO_2_/FiO_2_ **(A)**, AaDO_2_ **(B)**, EIT based end-expiratory lung volume (EELV) **(C)** and delta pressure to maintain normal PaCO_2_ **(D)** relationships for the low lung volume (*closed circles)*, medium lung volume (MLV) (*open diamonds*), and high lung volume (HLV) (*closed squares*) strategy. The variables at the openings- (*light gray colored*) and final (*dark gray colored)* pressure of the MLV and HLV intervention, as determined in Figure 2C, are indicated within the figures. Significant (one-way ANOVA) differences between the three interventions are only calculated at 120 min. Data are presented as mean and SD. *p < 0.05 in contrast to LLV, **p < 0.01 in contrast to LLV, ^‡^*p* < 0.01 in contrast to MLV.

**Figure 4:**
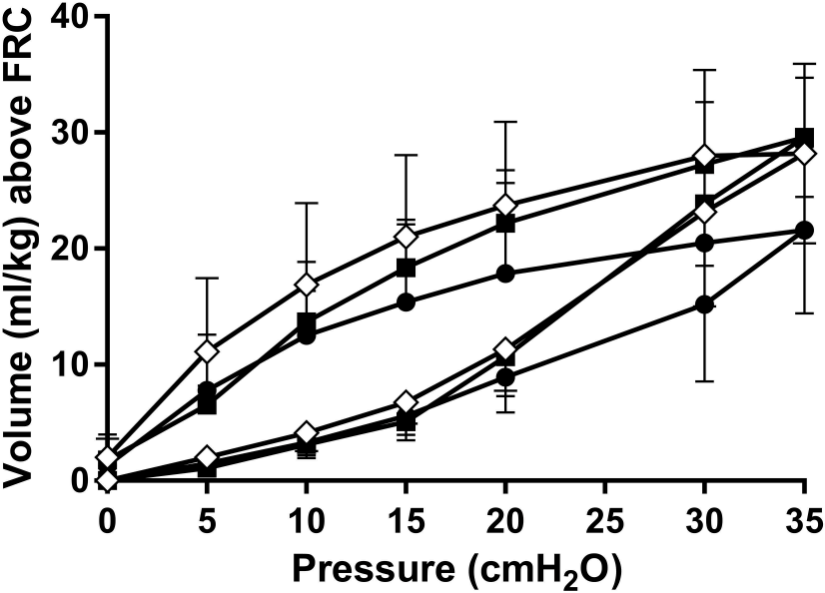
Static post mortem pressure-volume curves after 120 min of HFOV at a low lung volume (*solid circles*), medium lung volume (*open diamonds*), and high lung volume (*closed squares*) strategy using a super syringe technique. Data are presented as mean and SD.

Following the recruitment maneuver the benefits in SpO_2_/FiO_2_ and AaDO_2_ obtained during the MLV and HLV recruitment phase were maintained throughout the 120-min experimental period (Figures 3A,B). At all-time points measures of oxygenation were better in the HLV group compared to both MLV and LLV (*p* < 0.01). The same was true for the MLV compared with the LLV group (*p* < 0.01). In line, Figure 3C shows the same differences in EELV levels reached at CDPf, which was greater in the HLV group compared to only the LLV group (*p* < 0.01) and this effect did not change over time. The time to reach CDPo and CDPf and the level of EELV at these pressures were, respectively, longer and higher in the HLV group compared to the MLV group, but this did not reach statistical significance (Figure 2C). Regional EELV changed from the dependent to the non-dependent lung areas over time in the LLV group but this did not reach statistical significance, while in the MLV and HLV group this remained preferential tot the dependent lung areas (data not shown). The pressure amplitude needed to maintain the predefined PaCO_2_ was significantly higher in the LLV group than in the MLV (*p* < 0.05) and HLV groups (*p* < 0.01) (Figure 3D). There was no difference in the MLV and HLV pressure amplitude requirements. Although TLC during the post mortem pressure-volume curve was a mean 8.0 ± 3.4 ml/kg lower in the LLV than in the HLV group this difference did not reach statistical significance (Figure 4). C_max_ was greater in the MLV group in contrast to the HLV group but this didn't reach statistical significance (Table 2).

**Table 2:** Post mortem pressure volume curves, total lung protein, and histological injury data by gravity-dependent lung region.

**Ventilation strategy**	**UVC**			**LLV**			**MLV**			**HLV**		
**POST MORTEM VOLUME CURVE**												
TLC (ml/kg)	–			21.6 ± 7.2			28.2 ± 7.7			29.6 ± 5.2		
Cmax (ml/cmH_2_O/kg)	–			1.33 ± 0.79			1.88 ± 1.11			1.45 ± 0.52		
**BAL**												
Total protein (μg/μl)	204.7 ± 78.4			373.2 ± 161.3^$$^			303.2 ± 88.9^$$^			407.4 ± 133.7^$$^		
**HISTOLOGY**	**LOWER LR**	**MIDDLE LR**	**UPPER LR**	**LOWER LR**	**MIDDLE LR**	**UPPER LR**	**LOWER LR**	**MIDDLE LR**	**UPPER LR**	**LOWER LR**	**MIDDLE LR**	**UPPER LR**
**VILI DATA**												
Detached epithelial cells (no)	7.5 ± 6.4	4.9 ± 3.8	2.1 ± 1.0	24.1 ± 24.1	29.0 ± 21.1^$$^	28.9 ± 16.8^$$^	15.7 ± 12.0	18.0 ± 10.9	30.0 ± 11.4^$$^, ^*^	24.6 ± 11.6	30.2 ± 18.9^$$^	33.6 ± 19.4^$$^
Alveolar space (%)	70.6 ± 4.7	70.9 ± 6.4	74.2 ± 4.7	65.8 ± 4.0	64.0 ± 7.0	65.5 ± 7.3^$^	66.8 ± 11.0	64.6 ± 8.5	69.6 ± 6.6	71.5 ± 6.9	68.9 ± 5.2	67.6 ± 5.5
Lung tissue (%)	30.1 ± 4.7	29.9 ± 6.4	25.5 ± 4.7	34.2 ± 4.0	36.0 ± 7.0	34.5 ± 7.3^$^	33.2 ± 11.0	35.4 ± 8.5	30.4 ± 6.6	28.5 ± 6.9	31.1 ± 5.2	32.4 ± 5.5
Alveolar septa thickness (μm)	4.1 ± 0.6	3.8 ± 0.6	3.6 ± 0.2	7.5 ± 3.5^$^	9.3 ± 4.8^$$^	9.6 ± 6.3^$$^	6.1 ± 2.2	6.9 ± 2.9	7.1 ± 3.2	7.4 ± 2.6	7.4 ± 2.2	9.8 ± 2.7^$$^
Alveolar sac area (μm)	691.6 ± 99.9	709.8 ± 102.4	690.7 ± 74.0	709.2 ± 99.3	630.3 ± 79.4	595.4 ± 106.4^$^, ^*^	661.7 ± 75.0	610.4 ± 56.8^$^	670.0 ± 15.5	694.5 ± 106.9^$^	637.7 ± 91.7	614.7 ± 57.1
CoV of alveolar sac area	0.60 ± 0.08	0.63 ± 0.06	0.62 ± 0.08	0.68 ± 0.08	0.68 ± 0.09	0.56 ± 0.08^*^	0.64 ± 0.08	0.60 ± 0.07	0.63 ± 0.06	0.66 ± 0.09	0.62 ± 0.07	0.60 ± 0.05

*BAL, broncho-alveolar lavage; LR, lung region; LLV, low lung volume; MLV, medium lung volume; HLV, high lung volume; TLC, total lung capacity; UVC, unventilated controls; VILI, ventilator induced lung injury. All data are shown in mean and standard deviation*.

$ *p < 0.05 in contrast to UVC*.

$$ *p < 0.01 in contrast to UVC*.

* *p < 0.05 in contrast to lower lung region of the intervention*.

### Lung Injury Data

The total left lung protein levels were higher in all intervention groups compared with UVCs (all *p* < 0.01, Table 2), but between the groups there were no differences. Histological data showed that compared with the UVC group, all groups had more detached epithelial cells, as marker for damaged cells, in the non-dependent lung, and HLV and LLV in the middle third of the lung. In the LLV group the lower, middle, and upper lung region all had a thicker alveolar septum, indicative for change in lung morphology, compared to UVC's. Airway injury related markers showed only the upper lung region had a lower alveolar space, higher lung tissue, and lower alveolar saccular area compared with UVC's, while the middle region of the MLV group differed from UVC (higher alveolar saccular area), and in the HLV group the lower (higher alveolar saccular area) and upper (thicker alveolar septum) regions differed. Within the groups only the coefficient of variance of the alveolar sac area was significant lower in the upper region (*p* < 0.05) in contrast to the lower region in the MLV group.

**Figure 5:**
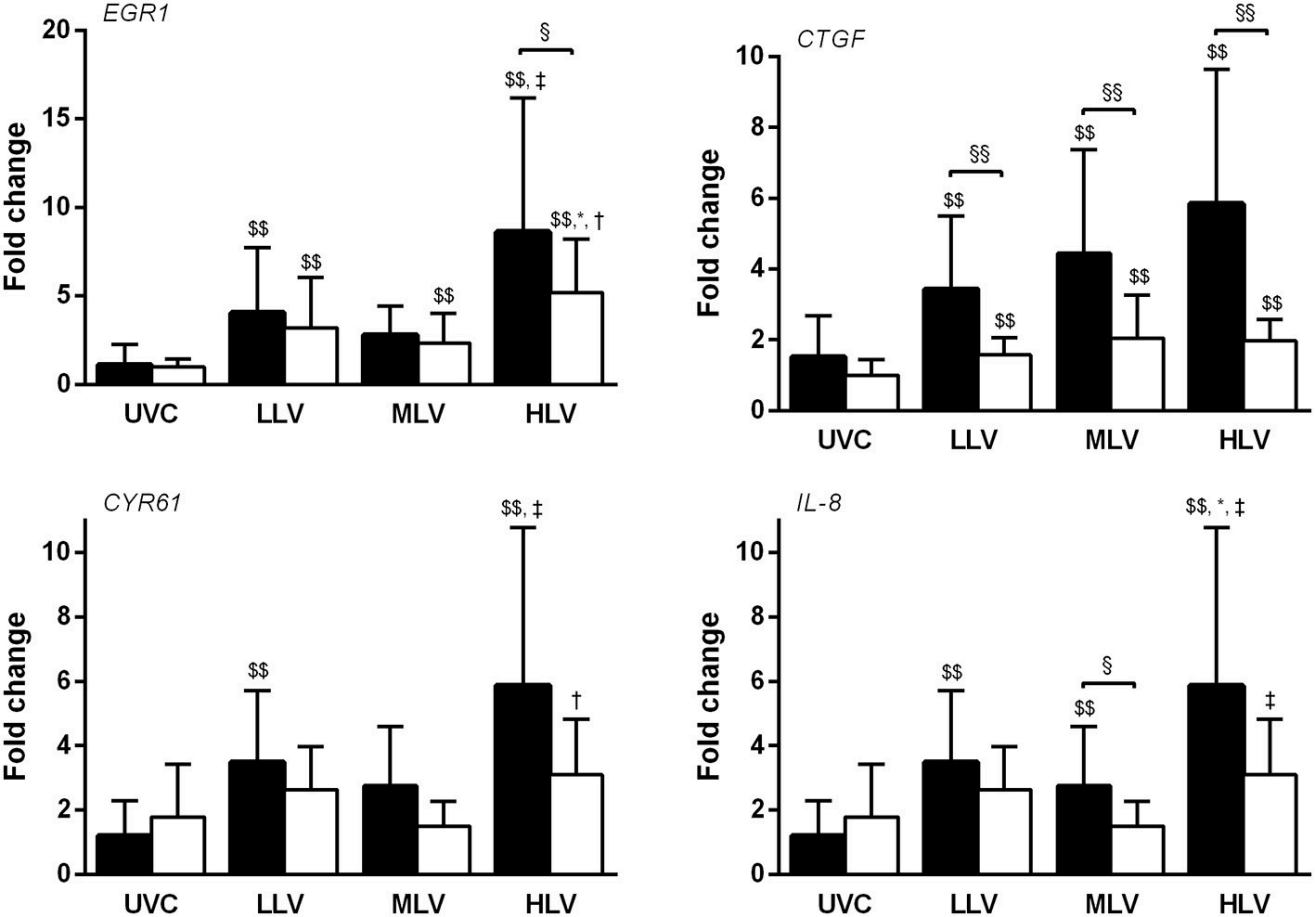
Gene expression of EGR1, CTGF, CYR61, and IL-8 mRNA in the non-dependent (*dark bars*) and dependent (*white bars*) lung region for the unventilated control (UVC), low lung volume (LLV), medium lung volume (MLV), and high lung volume (HLV) strategy in relation to reference gene GAPDH using the 2^−ΔΔCT^ method. Significant differences were calculated using one-way ANOVA with Turkey's *post-hoc* analyses. Data are presented as mean and SD. ^§^*p* < 0.05, ^§§^*p* < 0.01, ^$$^*p* < 0.05 in contrast to UVC, *p < 0.05 in contrast to LLV, ^†^*p* < 0.05 in contrast to MLV, and ^‡^*p* < 0.01 in contrast to MLV.

All interventional groups showed increase in mRNA expression of EGR1 and CTGF compared to UVC for both the dependent and non-dependent lung (all *p* < 0.01, Figure 5). Only the non-dependent lung demonstrated greater CYR61 and IL-8 expression (all ventilation groups) than UVC group. Between ventilation groups upregulation was higher in the HLV group, especially in the non-dependent lung, although this difference only reached statistical significance for EGR1, CYR61, and IL-8. Within group, generally mRNA expression was greater in the non-dependent lung compared to the dependent, reaching significance for CTGF (all ventilation groups), EGR1 (HLV only), and IL-8 (MLV only).

## Discussion

It has been suggested that optimizing EELV by reversing atelectasis using a recruitment maneuver is necessary to optimize gas exchange, improve lung mechanics, and attenuate lung injury during HFOV in preterm infants (4, 7, 8, 28). This is mainly based on animal experiments, in particular the study by McCulloch et al., and inference from clinical trial outcomes. However, the preclinical rationale is derived from studies of adult animals with saline lung lavage to induce surfactant deficiency. This does not mimic lung injury in respiratory distress syndrome following preterm birth in a developmentally immature lung (4). Furthermore, this study did not investigate to what extent lung volume should be optimized to improve outcomes. In our study we addressed these limitations by using a preterm lamb model of RDS and including two levels of lung recruitment.

Our study confirmed the observations of McCulloch et al. that lung recruitment is essential to ensure an oxygenation and ventilation benefit during HFOV (4). The LLV group was unable to maintain stable oxygenation and required significantly greater pressure amplitudes to achieve normocapnia compared to the two recruitment groups. The HLV group had superior oxygenation compared with the MLV group while ventilation was not different. Although the superior oxygenation is not an unexpected finding considering the design of the study, it does show that lung recruitment targeting a low FiO_2_ is feasible in a preterm lamb model of severe surfactant deficiency. This has also been shown in preterm infants with RDS (17).

Similar to clinical practice we used oxygenation as an indirect marker to assess lung volume recruitment (10, 17). Previous studies in animals and preterm infants have shown that oxygenation correlates well with EELV and thus can be used to guide lung recruitment during HFOV but lacks the precision to identify regional EELV differences that may increase injury risk (5, 10, 28, 39). For this reason changes in EELV were also measured. EIT is a novel method of directly measuring changes in lung aeration that also allows regional volume characteristics to be determined (10, 29, 30). To our knowledge, this is the first study to conclusively demonstrate that intended EELV response can be directly titrated from the target oxygen level during lung recruitment.

At birth the preterm lung is fluid-filled and exhibits different mechanics to the air-filled (aerated) lung. HFOV is not used clinically during the respiratory transition at birth, and our pilot study showed this was probably due to an insufficient driving pressure to facilitate aeration. It is for this reason we used an initial sustained inflation prior to HFOV, with a pressure-time approach we have previously validated in our lamb model (11, 14, 15, 19, 31). Notwithstanding this HFOV is still rarely used as the primary mode of support shortly after birth. We acknowledge this discrepancy in our study, but the choice was intentional. Ventilation modality influences lung injury and a brief period of conventional ventilation would have created lung injury that may have modulated the injury response during HFOV.

We assessed several parameters to determine the effect of lung recruitment on lung injury, but overall the differences between the LLV and both recruitment groups were modest and inconsistent. Importantly, lambs in the LLV had more pneumothoraces resulting in death compared to both recruitment groups and supports the need for lung recruitment. It also highlights the fallacy of considering all airleak as a complication of baro- or volutrauma, and the direct result of high applied pressure settings. Mathematical modeling have determined that high shear forces, which are considered the main cause of air leaks, are most prominent when zones of atelectasis are adjacent to zones of open lung units (32). Our study reaffirms that achieving lung recruitment creates more open lung units, and thus less shear forces within the lung. A finding that is substantiated by observational studies using a recruitment strategy in preterm infants (17, 33).

Injury induced deterioration of lung mechanics were assessed by reconstructing post mortem pressure-volume curves. These curves showed a tendency for a lower TLC and C_max_ in the LLV compared to the recruitment groups, although the magnitude of this effect was less impressive than the study of McCulloch et al. (4). Direct markers of lung injury were not clearly different between our lung volume groups. We can only speculate why these results did not support our hypothesis. Our study used extremely preterm lambs compared to previously healthy adult rabbits with induced lung disease, and previous preterm lamb studies have shown that 90 min is sufficient to detect differences in VILI histology scores (11, 13, 14, 24). The fundamental structural and functional developmental immaturity of the lungs in our lambs may result in lung injury, irrespective of the ventilation strategy applied. The frequent lack of difference in chronic lung disease rates in interventional respiratory support trials in preterm infants would support this. Our lamb model had very severe respiratory failure. This was intentional as in early preterm life HFOV is generally used as a rescue therapy for profound respiratory failure not responding to conventional ventilation. The magnitude of oxygen deficiency in our study though is higher than usually seen in clinical practice. It is possible that the apriori damage to the lung was so severe, that the ventilation strategy on lung injury was no longer a determining factor. In a less acutely sick population with less apriori lung injury, the impact of ventilation strategy on the total lung injury following 2 h of ventilation may be more pronounced. Similarly, 2 h of ventilation may have been too short to develop significant differences in lung injury between the groups.

To assess to what extent lung volume optimization is necessary for lung protection, we randomized the animals to two different levels of lung recruitment. In contrast to the intended benefit in oxygenation, markers of lung injury were not different between the HLV and MLV group. In fact, protein leakage and VILI-related genes, but not histology data, tended to be higher in the HLV than the MLV group. This finding was unexpected but intriguing. It is possible that the HLV and MLV did not achieve sufficient difference in the volume state of the lung. We hypothesize that our results also demonstrate the complexity of VILI in the preterm lung. VILI is rarely due to a single mechanism occurring throughout the lung. Increasing evidence has shown that the lung injury expression, is heterogeneous throughout the lung, often due to diametrically different mechanisms occurring at once, and rarely is injury potential truly ablated even if oxygen is optimized (12). The current suite of histological and early injury gene expression tools is designed to detect injury generally but lacks the sophistication to delineate multiple mechanisms occurring in specific lung regions. Thus, it is possible the mechanisms of injury differed between the two groups. For example the MLV may have had more regional atelectasis and the HLV transient overdistension. Further preclinical and clinical research is needed to determine the optimal balance of improving oxygenation and lung protection during lung recruitment maneuvers.

This study has some limitations not already addressed. Spontaneous breathing was suppressed during this study, for most infants lung volume is influenced by both mean airway pressure changes and the presence or absence of spontaneous respiratory effort. Spontaneous breathing may also have altered the injury profiles. We did not assess the impact of OLV on cerebral- or pulmonary blood flow while these data could have altered outcomes (34). However, heart rate and mean blood pressure did not differ between groups. Our ventilation strategy was not entirely consistent with clinical practice. The frequency, power and time per pressure step was based on clinical application of HFOV in infants (9, 10, 17, 35) and our previous lamb study (15). We did not administrate (prophylactic) endotracheal surfactant to emulate the type of infants receiving HFOV in most NICU. Surfactant significantly alters mRNA and histological injury expression in the preterm lamb, and may have masked differences between the recruitment strategies. This should be considered in translating our results (36). Furthermore, it is no longer advocated that prophylactic endotracheal surfactant administration is the best line treatment. Also it is suggested that first recruiting the lungs and give surfactant to only a selected patient category is more efficient (37).

In conclusion, our study confirms the importance of lung recruitment to optimize gas exchange during HFOV in the preterm model of RDS. However, the benefit of recruitment on lung injury is less evident compared to adult models of lung injury. This finding may, in part, explain why clinical trials comparing HFOV to CMV in preterm infants, have produced less favorable effect of HFOV on short term pulmonary outcomes of lung protection than (adult) animal models. It is encouraging to see that long term pulmonary outcomes seem to be better in HFOV treated infants compared to CMV (38). We were unable to confirm that optimizing oxygenation during lung recruitment also reduced lung injury. It therefore remains unclear what target FiO_2_ should be used during oxygenation guided lung recruitment during HFOV in preterm infants.

## Author Contributions

MM, DT, and AvK contributed to the study conception and design. MM, KM, EP, RO, PP-F, AR, and DT contributed to acquisition of data. MM, DT, and AvK analyzed and interpreted data and drafted the manuscript. All authors revised the manuscript for important intellectual content and approved the final version of the manuscript.

### Conflict of Interest Statement

AW is an employee of Swisstom AG, the company producing commercial adult EIT and research devices. Swisstom customized the EIT hard- and software for the preterm lamb model used in this report. AW helped interpret the EIT data and revised the manuscript. All EIT hard- and software used in this study was purchased by the Murdoch Children's Research Institute without restriction. The remaining authors declare that the research was conducted in the absence of any commercial or financial relationships that could be construed as a potential conflict of interest.
