# Supplementary material for: Lung Recruitment Strategies During High Frequency Oscillatory Ventilation in Preterm Lambs
Source: Front Pediatr. 2019 Jan 22;6:436. doi: 10.3389/fped.2018.00436 (PMC6349831; doi:10.3389/fped.2018.00436)
Supplement: Supplementary file 2 [file Data_Sheet_2.doc]

**Supplementary material 2**

**Individual pressure – SpO_2_/FiO_2_, AaDO_2_ and end expiratory lung volume curves during the high lung volume recruitment strategy**

**
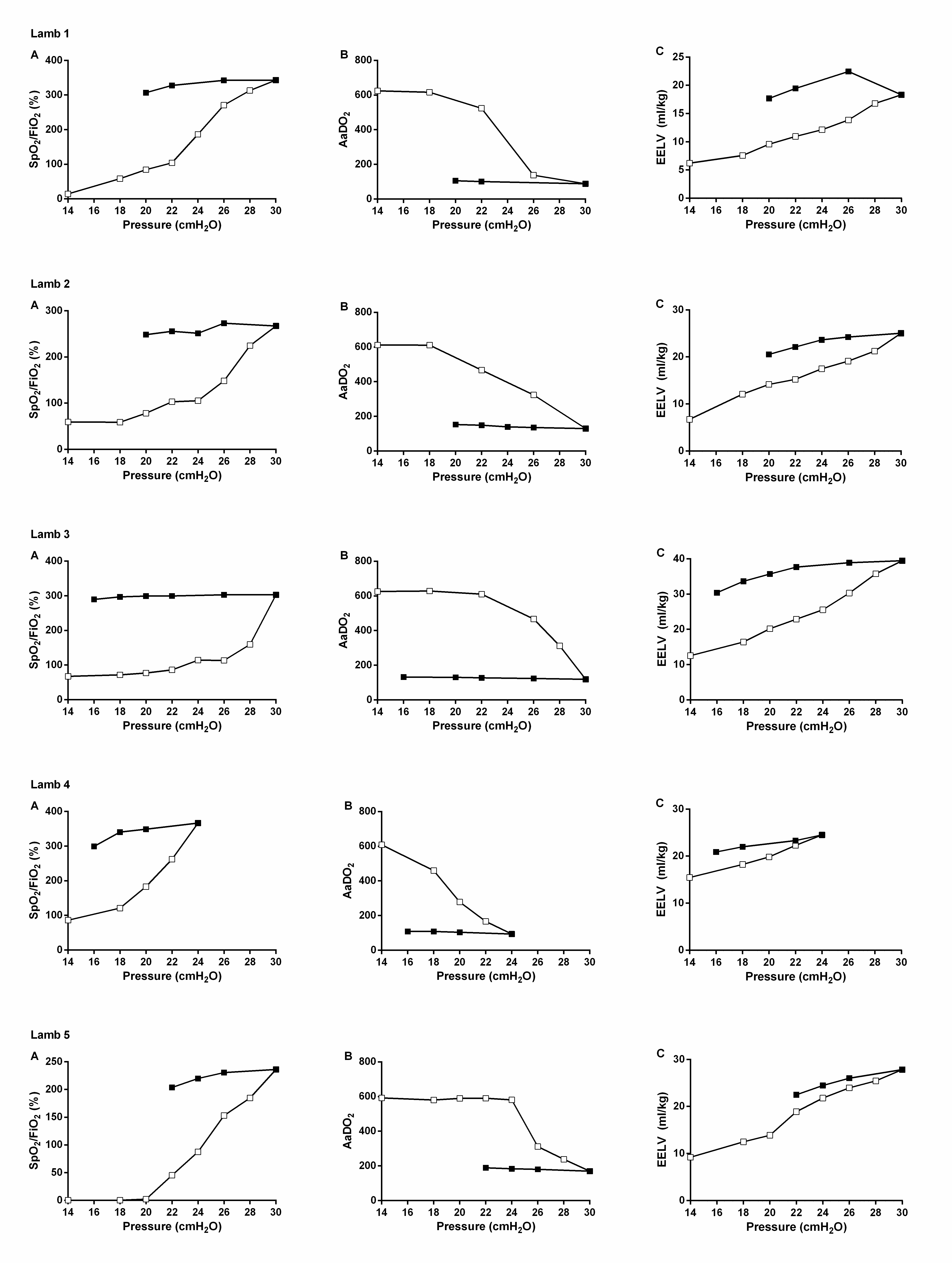

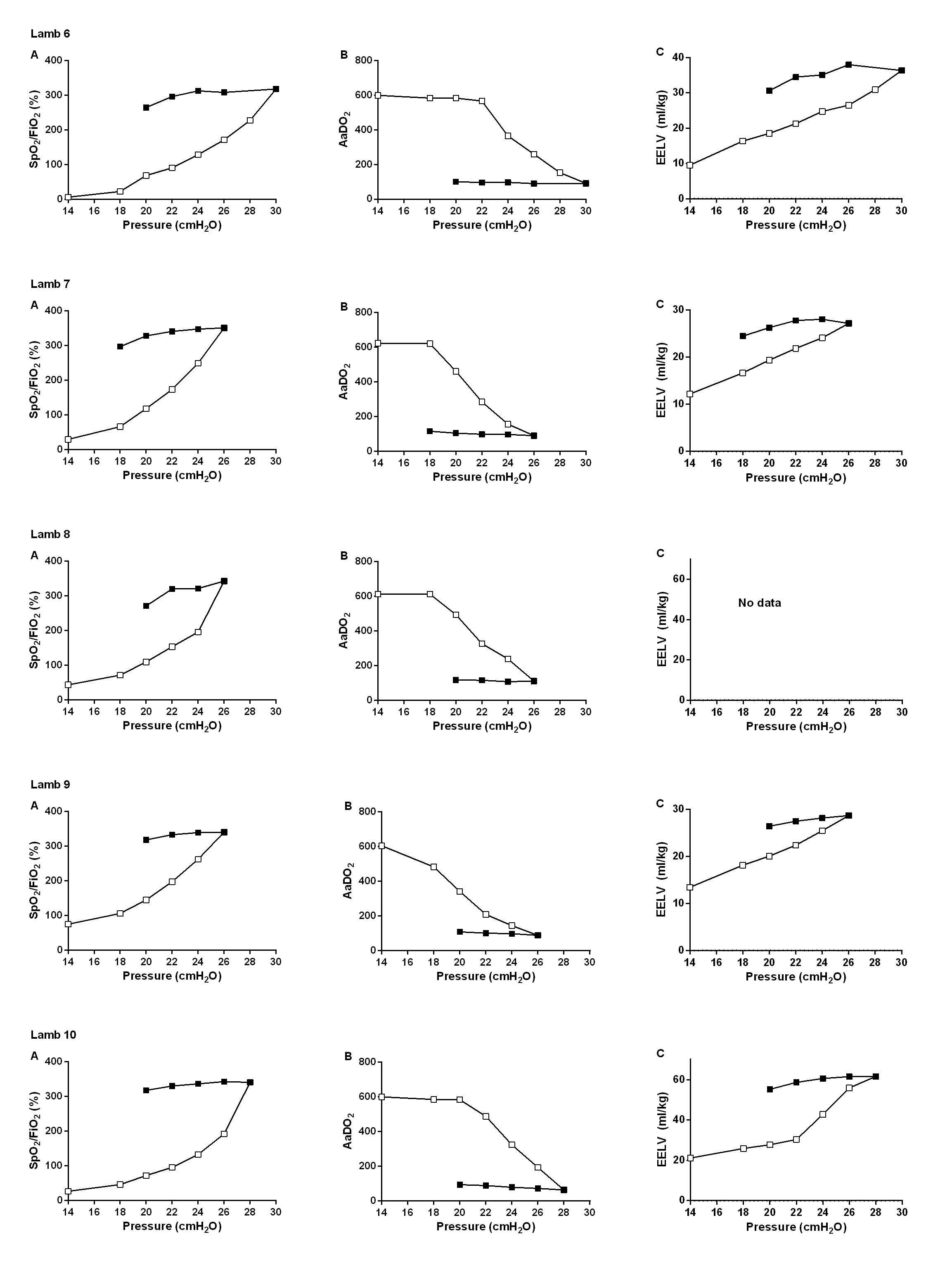
**
